# High-Quality Perovskite Films Enabled by Solution-Processed Vacuum Evaporation for Flexible PIN-Type X-Ray Detectors

**DOI:** 10.3390/molecules31071123

**Published:** 2026-03-29

**Authors:** Yali Wang, Hongjun Mo, Sai Huang, Haonan Li, Xinyang Huang, Weiguang Yang

**Affiliations:** 1 School of Materials Science and Engineering, Shanghai University, Shanghai 200444, China; w1770495171@163.com (Y.W.);; 2 Zhejiang Institute of Advanced Materials, Shanghai University, Jiashan 314113, China; 3 State Key Laboratory of Advanced Special Steels, Shanghai 200444, China

**Keywords:** metal halide perovskite, flexible X-ray detector, composition engineering

## Abstract

Flexible X-ray detectors have emerged as a promising technology for portable medical imaging and wearable electronics, yet their manufacturing remains constrained by the competing requirements of device performance, mechanical conformability, and production scalability. Conventional solution-based deposition methods fail to yield high-quality perovskite thick films with uniform morphology, while vacuum evaporation techniques are limited by exorbitant operational costs and low throughput. Herein, we report an optimized solution-processed vacuum evaporation strategy that enables the fabrication of high-quality perovskite films (~1 μm thick) on flexible polyethylene naphthalate (PEN) substrates at a low processing temperature of 100 °C. By incorporating tailored additives into the precursor solution and precisely modulating the vapor-phase conversion kinetics, we achieved significant improvements in film density, crystallinity, and morphological uniformity. Systematic investigations were conducted to elucidate the structure–property relationships across three material systems: pure methylammonium lead iodide (MAPbI_3_), halogen-doped methylammonium lead iodide-bromide (MAPb(IBr)_3_), and synergistic cation-halogen engineered cesium-methylammonium lead iodide-bromide (CsMAPb(IBr)_3_). The optimized flexible PIN-type X-ray detector based on CsMAPb(IBr)_3_ exhibited exceptional performance metrics, including a dark current density as low as 5.2 nA cm^−2^ and an X-ray sensitivity of up to 1.43 × 10^4^ μC·Gy_air_^−1^·cm^−2^. Remarkably, the device retained over 95% of its initial performance after 400 bending cycles with a bending radius of 6 mm, demonstrating outstanding mechanical robustness and operational durability. This work establishes a viable, cost-effective technical route for the scalable production of high-performance flexible X-ray detectors, addressing critical challenges in the advancement of next-generation portable imaging technologies.

## 1. Introduction

Metal halide perovskites (MHPs) are a class of semiconductor materials that exhibit outstanding optoelectronic properties [1,2,3]. Their material characteristics—including high X-ray absorption coefficients (10^5^–10^6^ cm^−1^), tunable bandgap structure, long carrier diffusion length and high defect tolerance—make them highly suitable for next-generation low-dose, high-sensitivity X-ray detection applications [4,5,6]. Compared to conventional direct-conversion semiconductors, perovskites demonstrate superior performance in key metrics. For instance, at an energy of 60 keV, the mass attenuation coefficient of perovskites (~4.5 cm^2^/g) exceeds that of amorphous selenium (α-Se, ~3.8 cm^2^/g) [7] and is comparable to cadmium zinc telluride (CdZnTe, ~4.7 cm^2^/g) [8]. Furthermore, the carrier mobility-lifetime (μτ) product of perovskites (>10^−3^ cm^2^/V) is orders of magnitude higher than that of α-Se (<10^−6^ cm^2^/V) [9], enabling significantly higher charge collection efficiency under low-dose radiation [10]. While α-Se detectors remain the gold standard for mammography due to their excellent spatial resolution and direct-conversion principle, they require high bias voltages (>10 V/μm) and suffer from poor thermal stability and weak X-ray stopping power. Conversely, although CdZnTe possesses strong X-ray absorption, its widespread application is hindered by high manufacturing costs, compositional non-uniformity, and Te-related defects [11]. In contrast, perovskite materials synergize high sensitivity, low-voltage operation, and cost-effective processing with inherent mechanical flexibility [12]. Furthermore, compared to traditional semiconductor materials, perovskite films can be synthesized via low-temperature solution processing, enabling the development of large-area, low-cost, and flexible devices [13,14,15]. Despite these advantages, intrinsic instabilities—such as ion migration and moisture sensitivity—remain primary challenges for their commercialization [16,17]. With the rise of wearable electronics and portable medical imaging systems, the development of flexible X-ray detectors has become a significant research frontier [18,19]. However, the fabrication of perovskite films for direct-detection X-ray detectors presents a critical technical trade-off among material crystalline quality, fabrication cost, and device mechanical flexibility.

The performance metrics of direct-detection X-ray detectors, such as dark current density and signal-to-noise ratio, are highly dependent on the crystalline quality and thickness of their absorption layer (intrinsic layer) [20,21]. To achieve high crystalline quality, detectors based on perovskite single crystals have been developed; these exhibit outstanding performance, including extremely low dark current density (nA cm^−2^) and high sensitivity, attributable to their exceptionally low internal defect density [22,23,24]. For instance, FAPbBr_3_ single crystals have shown bulk specific sensitivity of 7.28 C·Gy^−1^·cm^−3^ at 0 V, demonstrating ultra-high operational stability over 26 days of uninterrupted X-ray exposure with negligible signal loss [25]. However, single-crystal fabrication methods are typically costly and yield-limited; furthermore, the inherent rigidity of single crystals renders them unsuitable for flexible device manufacturing [23,26]. Conversely, solution processing (e.g., spin-coating) offers simplicity, low cost, and compatibility with flexible substrates, yet it struggles to produce high-quality micrometre-scale films. Although techniques such as doctor-blade coating, inkjet printing, and screen printing have been employed to increase film thickness [27,28,29], these methods frequently introduce surface roughness and solvent residue issues. During thick-film deposition, rapid solvent evaporation frequently induces uncontrolled crystallization processes, resulting in films with high-density grain boundaries and pinholes [29]. These defects form leakage pathways, significantly elevating device dark currents (up to the μA cm^−2^) and acting as charge trap centers that reduce carrier collection efficiency, thereby causing severe signal tailing [21].

To meet the demands of flexible detectors, various innovative fabrication techniques and material designs have been proposed. For instance, large-area flexible X-ray detectors based on 2D/3D perovskite thick films prepared via drop-casting have achieved sensitivities of 232 μC·Gy_air_^−1^·cm^−2^, with optimized low-temperature deposition and annealing processes ensuring compatibility with flexible substrates [30]. Flexible single-microwire X-ray detectors have also demonstrated ultra-high sensitivity, which is of great significance for portable radiation detection systems [31]. Another innovation involves utilizing ultra-thin (500 nm) wide-bandgap metal halide perovskite films to construct flexible X-ray detectors, achieving sensitivities as high as 308 μC Gy^−1^ cm^−2^ while exhibiting excellent self-powered capability and operational stability under prolonged X-ray exposure [32]. Significant progress has also been made in addressing the intrinsic ion migration issues of perovskite materials. For example, CsPbBr_3_ single crystals prepared through the atmosphere-controlled edge-defined film-fed growth (EFG) method effectively suppress ion migration, thereby enhancing X-ray detection and imaging performance [33]. Furthermore, in (Br-EA)_2_PbBr_4_ perovskite single crystals, enhancing the dipole moment via A-site cation halogen substitution has been proven to inhibit ion migration and improve X-ray detection performance, while also exhibiting novel characteristics such as additional dipole-response currents [34].

As an alternative, high-vacuum thermal evaporation enables the preparation of high-quality, uniform films [35]. However, its high equipment costs and slow deposition rates limit its application in low-cost, large-scale production [36].

To address these issues, a hybrid solution-processed vacuum evaporation process offers an alternative solution. This approach divides thin-film fabrication into two distinct steps. First, a uniform layer of inorganic precursor (e.g., PbI_2_) is deposited via a solution-based method. Subsequently, a gas-phase reaction with organic components (methylammonium iodide, MAI), followed by thermal annealing, converts the precursor into a high-quality perovskite film [4]. This method has been successfully applied to fabricate high-efficiency perovskite solar cells on rigid substrates, effectively decoupling the nucleation and growth processes to enhance film morphology and crystallinity [37]. The advantage of the PIN junction lies in its built-in electric field, which effectively separates X-ray-induced electron-hole pairs while significantly inhibiting carrier injection (particularly holes) from the electrodes into the semiconductor layer, thereby reducing the dark current [38]. This charge separation mechanism, combined with the suppression of space-charge-limited current, enables the PIN structure to achieve a higher signal-to-noise ratio and a lower limit of detection in low-dose X-ray sensing. However, several technical challenges must be addressed when adapting this process for flexible PIN-type X-ray detectors. First, the conversion process is required to be completed at low temperatures (≤100 °C) due to the limited thermal tolerance of flexible substrates. Second, for micrometre-scale films, it is critical to ensure that gaseous MAI molecules can fully diffuse to the base of the PbI_2_ film to achieve complete chemical conversion and prevent PbI_2_ residues. Third, to meet the low leakage current requirements of PIN junction devices, the prepared perovskite intrinsic layer must be sufficiently dense to effectively block charge leakage. Recent advancements have focused on innovative strategies to enhance device performance, such as defect passivation in Ruddlesden–Popper perovskites to develop high-performance flat-panel X-ray detectors [39], and the suppression of ion migration using atmosphere-controlled edge-defined film-fed growth (EFG) methods for CsPbBr_3_ single crystals [37]. In this study, we systematically investigated the processing–structure–performance relationship of perovskite films fabricated via an optimized solution-processed vacuum evaporation strategy. By correlating the vapor-phase conversion kinetics (processing) with the resulting film crystallinity and grain morphology (structure), we achieved a fundamental understanding of the charge transport and trapping mechanisms that govern X-ray detection sensitivity and dark current. Building upon these developments, our work focuses on achieving high-quality polycrystalline films through a low-temperature hybrid process.

**Figure 1:**
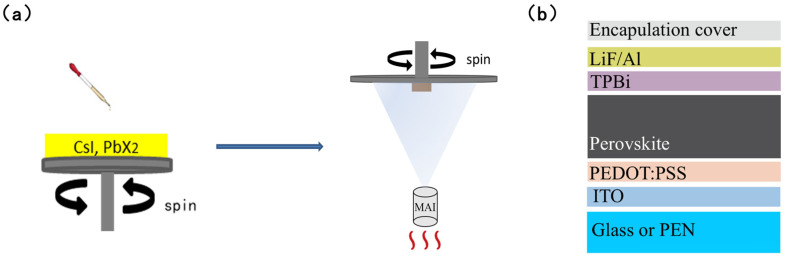
(**a**) Schematic of the solution-processed vacuum evaporation preparation process. (**b**) Schematic diagram of the structure of the PIN-type X-ray perovskite detector.

This work aims to address the aforementioned technical challenges by systematically optimizing the deposition process to fabricate high-performance flexible X-ray detectors. The schematic of the solution-processed vacuum evaporation preparation process and the PIN-type X-ray perovskite detector structure are shown in Figure 1 Three types of precursor solutions—pure PbI_2_, PbI_2_/PbBr_2_ mixtures, and CsI/PbI_2_/PbBr_2_ mixtures—were prepared and deposited onto flexible polyethylene naphthalate (PEN) substrates by regulating the spin-coating parameters. These solution-processed inorganic films served as templates for the subsequent hybrid evaporation process. By controlling the MAI evaporation temperature and annealing duration, the complete conversion of 1-μm-thick PbI_2_ films was achieved at 100 °C. To further benchmark the performance of our (Cs_0.05_MA_0_._95_)Pb(I_0.8_Br_0.2_)_3_ film, we have provided a comprehensive comparison with previously reported hybrid deposition methods and performance metrics in Table 1. Compared to traditional blade-coating, screen-printing, and spray-coating techniques, our solution-processed vacuum evaporation method yields high-quality films with superior sensitivity even at zero bias. This performance is particularly notable given the relatively small thickness (1 μm), highlighting the exceptional charge collection efficiency and reduced defect density achieved through Cs^+^/Br^−^ co-doping. Utilizing these high-quality films, flexible PIN-type X-ray detectors were constructed [40]. The devices exhibited a dark current density of 5.2 nA cm^−2^ and an X-ray sensitivity of 1.43 × 10^4^ μC·Gy_air_^−1^·cm^−2^. Mechanical performance testing revealed that after 400 bending cycles (bending radius 6 mm), critical parameters including dark current and sensitivity exhibited less than 5% variation. These results demonstrate that the optimized solution-processed vacuum evaporation process is an effective method for fabricating high-quality flexible perovskite films, providing a technological foundation for developing low-cost, large-area flexible X-ray imaging systems.

## 2. Results and Discussion

**Figure 2:**
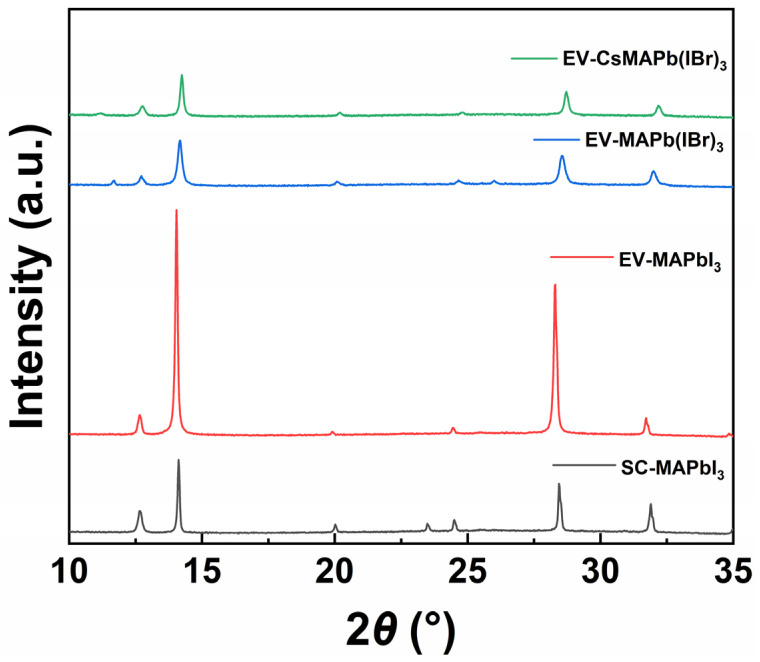
XRD patterns of perovskite films prepared by different processes and compositions, where SC denotes spin-coating and EV denotes solution-processed vacuum evaporation.

As shown in Figure 2, the MAPbI_3_ film prepared via the conventional spin-coating method exhibits diffraction peaks corresponding to the perovskite (110) and (220) crystal planes near 14.1° and 28.4°, respectively. In contrast_,_ the EV-MAPbI_3_ exhibited markedly different XRD patterns. The intensities of the (110) and (220) diffraction peaks were significantly enhanced, with noticeably narrower peak widths, indicating improved crystallinity and larger grain sizes. Both Br-doped films and Cs^+^/Br^−^ co-doped films retained strong and sharp perovskite diffraction peaks, confirming their high crystalline quality. Concurrently, a systematic shift in the (110) main diffraction peak towards higher 2*θ* angles was observed in the doped films compared to the pure MAPbI_3_ film. This shift is attributed to a reduction in the lattice constant resulting from the substitution of smaller Br^−^ ions for some I^−^ ions, providing evidence for successful ion incorporation. Compared to the film doped solely with Br^−^ (peak position 14.18°), the (Cs_0.05_MA_0.95_)Pb(I_0.8_Br_0.2_)_3_ film exhibits a further shift in its (110) diffraction peak to 14.24°. This additional shift arises from the smaller radius of Cs^+^ ions relative to methylammonium cations (MA^+^). The synergistic incorporation of Cs^+^ and Br^−^ induces further lattice constant contraction, causing the diffraction peak to shift towards larger angles. Notably, a weak diffraction peak at approximately 12.6° is observed in the EV-processed films, which is indexed to the (001) plane of residual PbI_2_. This suggests a slight incomplete conversion at the film-substrate interface due to the diffusion limits of MAI vapor through the 1-μm-thick layer. However, such trace amounts of PbI_2_ are known to provide grain boundary passivation [49], which contributes to the suppressed non-radiative recombination and the low dark current density observed in our EV-type detectors. To further validate the phase purity and elemental homogeneity, EDS elemental mapping (Figures S4 and S5) was performed, revealing a uniform distribution of Cs, Pb, I, and Br across the film without detectable segregation. Quantitative analysis (Table S2) confirmed a Cs content of 0.43 at%, consistent with the successful incorporation of the A-site cation.

**Figure 3:**
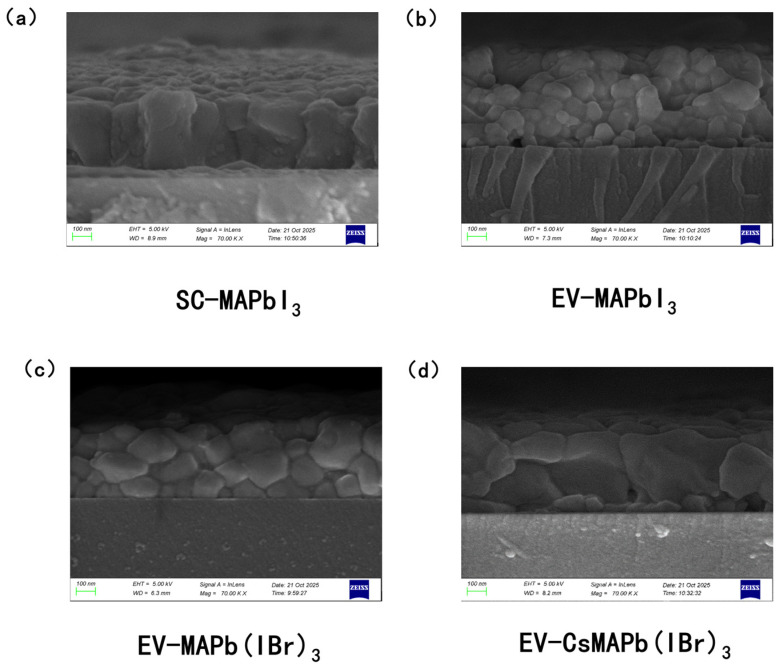
SEM images of perovskite films with different processes and compositions. (**a**) SC-MAPbI_3_ film; (**b**) EV-MAPbI_3_ film; (**c**) EV-MAPb(I_0.8_Br_0.2_)_3_ film; (**d**) EV-(Cs_0.05_MA_0.95_)Pb(I_0.8_Br_0.2_)_3_ film.

The effects of fabrication processes were compared, both based on the same PbI_2_ precursor film, on the morphology of the resulting perovskite films. MAPbI_3_ films prepared via the conventional spin-coating method exhibited a columnar growth morphology. This is attributed to the solvent in the MAI solution providing a transient liquid-phase environment, which promotes oriented grain growth and sufficient annealing. Conversely, the EV-MAPbI_3_ films exhibited a stacked grain structure. This disparity stems from the diffusion-limited nature of the solid-state transformation; the rapid reaction driven by high MAI evaporation rates, coupled with a lack of solution-mediated rearrangement, triggers multiple discontinuous nucleation events within the film. To overcome the kinetic limitations in solid-state transformation, composition engineering was introduced by doping Br^−^ or Cs^+^ ions into the PbI_2_ solution. As shown in the SEM images (Figure 3), significant enlargement of the grain sizes was observed in the doped MAPb(I_0.8_Br_0.2_)_3_ and (Cs_0.05_MA_0.95_)Pb(I_0.8_Br_0.2_)_3_ films. This improvement primarily stems from the synergistic introduction of both Br^−^ and Cs^+^ ions, which elevates the crystallization energy barrier and retards the solid-state transformation rate. The Goldschmidt tolerance factor (t) is a crucial geometric parameter used to predict the stability of perovskite crystal structures. For stable perovskite formation, the tolerance factor typically falls within the range of 0.8 to 1.0. [9]. Concurrently, Cs^+^ ion doping optimizes the lattice tolerance factor, thereby reducing nucleation density [50]. This compositional engineering strategy successfully modulates the crystallization kinetics of the solid-state transformation process, providing an effective pathway for obtaining high-quality, large-grain films within the spin-coating-evaporation system.

**Figure 4:**
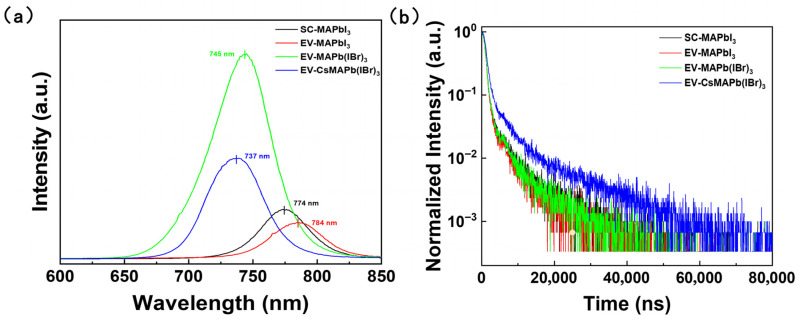
(**a**) Steady-state photoluminescence (PL) spectra of perovskite films prepared by different processes and compositions. (**b**) Time-resolved photoluminescence (TRPL) spectra.

To elucidate the effects of the fabrication process and compositional engineering on the intrinsic optoelectronic quality of the films, both steady-state photoluminescence (PL) and time-resolved photoluminescence (TRPL) spectroscopy were conducted. The results are presented in Figure 4 and summarized in Table 2.

For pure MAPbI_3_ films, a direct comparison reveals that the conventional spin-coated (SC) sample exhibits superior optoelectronic characteristics. It demonstrates a higher PL intensity and a longer average TRPL lifetime (874.63 ns) compared to the hybrid evaporation (EV) sample (795.71 ns). This suggests that for the pure iodide system, the one-step SC process may offer better control over the crystallization kinetics. Specifically, EV-MAPbI_3_ exhibits a slight PL red-shift (784 nm) compared to SC-MAPbI_3_ (774 nm). This is attributed to its superior crystallinity and reduced lattice strain, which allows the emission to approach the intrinsic band-edge of MAPbI_3_, whereas the SC-processed films often suffer from lattice distortion-induced bandgap enlargement due to rapid crystallization.

However, the potential of the hybrid EV process is realized through compositional engineering. A significant improvement is achieved through the introduction of bromine to form EV-MAPb(IBr)_3_. The PL emission peak blueshifts to ~745 nm, confirming Br incorporation, and more strikingly, the PL intensity increases dramatically by over five-fold. This pronounced PL enhancement, coupled with a marginal increase in carrier lifetime to 1978 ns, strongly indicates that a large number of non-radiative defect centers are effectively passivated by Br^−^ incorporation, thereby substantially improving the film’s radiative efficiency and crystalline quality.

Theoretically, the carrier lifetime (τ) is governed by the radiative (k_r_) and non-radiative (k_nr_) recombination rates according to the relation 1/τ = k_r_ + k_nr_. In polycrystalline perovskites, k_nr_ is predominantly determined by the trap-state density, where defects act as recombination centers that provide non-radiative pathways for charge carriers [4]. By suppressing these trap states through Br-incorporation, the non-radiative recombination is significantly inhibited, leading to the observed prolongation of carrier lifetime and enhancement in PL intensity.

The most profound optimization is achieved with synergistic Cs^+^/Br^−^ co-doping. A remarkable average lifetime of 3497.34 ns is exhibited by the (Cs_0.05_MA_0.95_)Pb(I_0.8_Br_0.2_)_3_ film, representing a fourfold increase over its Cs-free counterpart (MAPb(I_0.8_Br_0.2_)_3_). Analysis of the TRPL decay parameters reveals that the weight fraction of the slow decay component (τ_2_) increases markedly from ~23% to 61.22%, confirming that non-radiative recombination centers within the film bulk are significantly reduced by Cs^+^ incorporation. The dominance of the slow decay component (τ_2_) in the Cs/Br co-doped film suggests that the bulk trap states are effectively passivated, allowing carriers to persist longer before recombination. While the Cs/Br co-doped film exhibits a significantly extended TRPL lifetime of 3497.34 ns, suggesting reduced bulk non-radiative recombination, a noticeable quenching in steady-state PL intensity is observed. This suggests that while bulk quality is improved, surface recombination or other non-radiative pathways may still play a role under steady-state conditions.

To quantitatively evaluate the trap-state density (Nt), space-charge-limited current (SCLC) measurements were performed on hole-only devices (Figure S7). The trap-filled limit voltage (V_TFL_) was determined from the kink point in the log–log I-V curves. The V_TFL_ values were found to be 2.32 V, 0.81 V, and 0.26 V for the EV-MAPbI_3_, EV-MAPb(IBr)_3_, and EV-CsMAPb(IBr)_3_ films, respectively. The significantly lower V_TFL_ in the Cs/Br co-doped sample indicates a substantial reduction in Nt, which is highly consistent with the prolonged carrier lifetimes observed in TRPL and justifies the superior charge collection efficiency in the final detectors.

**Figure 5:**
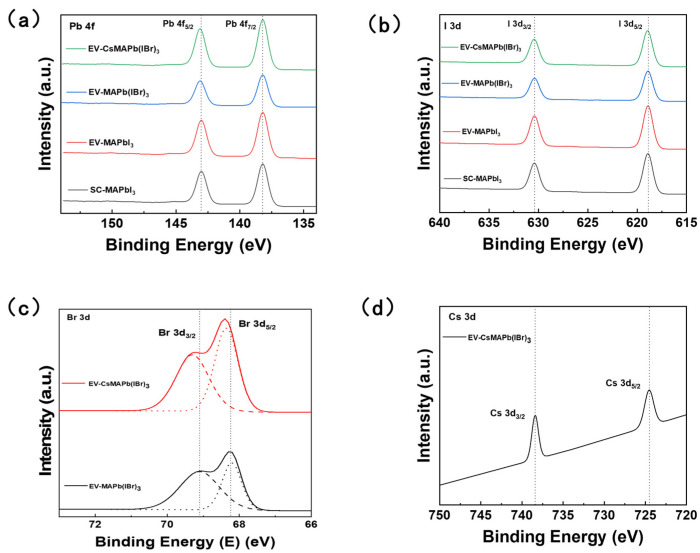
(**a**) Pb 4f, (**b**) I 3d, (**c**) Br 3d, and (**d**) Cs 3d XPS spectra of perovskite films with different processes and compositions. The vertical dotted lines are provided as visual guides to indicate peak positions and shifts.

To highlight the advantages of the component engineering strategy, comparative XPS analysis was conducted on the prepared perovskite films, as shown in Figure 5. Within the spectrum of the MAPb(I_0.8_Br_0.2_)_3_ sample, a characteristic Br 3d peak was distinctly observed at approximately 68.5 eV, confirming the successful incorporation of bromine. Notably, the Pb 4f spectrum exhibited subtle changes upon bromine introduction: while the Pb 4f_7/2_ peak position remained essentially unchanged at ~138.2 eV, the Pb 4f_5/2_ peak showed a slight positive shift of ~0.1 eV in the MAPb(I_0.8_Br_0.2_)_3_ and (Cs_0.05_MA_0.95_)Pb(I_0.8_Br_0.2_)_3_ samples compared to the pure iodide counterparts. This minor divergence in the spin–orbit doublet suggests a subtle modification of the Pb electronic environment induced by the more electronegative bromine, though the effect on the core Pb^2+^ states is limited. This confirms that bromine incorporation results in a homogeneous MAPb(I_0.8_Br_0.2_)_3_ mixed-halide lattice rather than a physical mixture of separate phases. Furthermore, in the (Cs_0.05_MA_0.95_)Pb(I_0.8_Br_0.2_)_3_ film, a distinct Cs 3d_5/2_ characteristic peak at approximately 724.5 eV is observed, providing direct chemical evidence for the successful incorporation of Cs^+^ ions. While the Pb 4f (~138.2 eV) and I 3d (~619.3 eV) peaks exhibited no significant chemical shift following the introduction of Cs^+^, the Br 3d spectrum displayed a discernible change in peak profile and position (Figure 5c). This variation in the Br 3d binding energy is attributed to the lattice contraction and the altered local electrostatic environment induced by the incorporation of the smaller Cs^+^ cations, further confirming the successful formation of the mixed-cation lattice. Despite this local modulation, the core chemical state of Pb^2+^ remains preserved without reduction to metallic lead. These results confirm that the A-site cation engineering strategy enables precise control of perovskite composition while effectively preserving the chemical state and coordination environment stability of its inorganic framework. This chemical stability provides a fundamental explanation for the suppressed non-radiative recombination and the substantial prolongation of carrier lifetimes observed in TRPL measurements. SEM analysis visually demonstrates that composition engineering (particularly the introduction of Cs^+^) markedly increases the grain size of the film. Typically, larger grains correspond to a lower density of grain boundaries, thereby reducing defect density at these interfaces. However, a more intricate intrinsic mechanism is revealed by the PL and TRPL results. While larger grains reduce the macroscopic grain boundary density, the carrier lifetime is predominantly determined by the defect density within the grain bulk.

Both DFT calculations and experimental studies have demonstrated that the introduction of Br^−^ can enhance lattice stability and suppress the formation of intrinsic defects, thereby reducing the trap-state density and prolonging carrier lifetime [50]. Simultaneously, the incorporation of Cs^+^ effectively releases lattice strain and stabilizes the perovskite framework, further inhibiting ion migration and reducing point defects. The subtle shifts in Pb 4f peaks and the variations in Br 3d peaks observed in XPS results provide atomic-level evidence of the optimized local chemical environment and lattice structure induced by Cs^+^/Br^−^ co-doping [34]. The introduction of Cs^+^ enhances bulk quality by stabilizing the lattice and reducing point defects (as demonstrated by TRPL), whereas PL quenching primarily reflects the dynamic instability of mixed halide systems under illumination.

**Figure 6:**
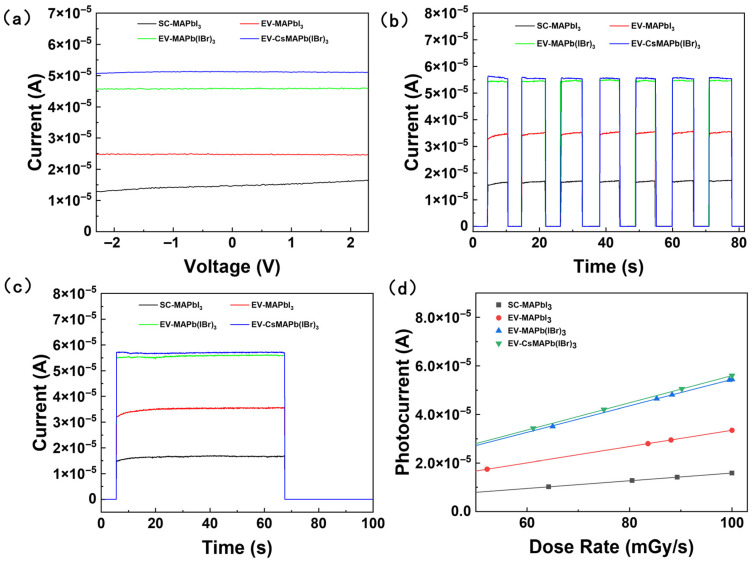
(**a**) I-V characteristics of perovskite devices; (**b**) dynamic response to X-ray irradiation; (**c**) continuous irradiation for 60 s; (**d**) linear behavior of photocurrent as a function of incident dose rate. for perovskite devices on glass substrates. All devices were encapsulated with PDMS to isolate moisture and oxygen.

To systematically evaluate the comprehensive impact of the fabrication processes and composition engineering on the optoelectronic performance of the devices, steady-state photocurrent–voltage (I-V) measurements were conducted on devices fabricated on rigid (Figure 6a) and flexible (Figure S1a) substrates. As shown in Figure 6a, compared to conventional spin-coated MAPbI_3_ devices (photocurrent ~1.46 × 10^−5^ A), devices fabricated using the optimized solution-processed vacuum evaporation method demonstrated significantly enhanced performance (~2.49 × 10^−5^ A). Furthermore, a substantial increase in photocurrent was achieved through compositional engineering via Br^−^ and Cs^+^ incorporation. The optimal device, (Cs_0.05_MA_0.95_)Pb(I_0.8_Br_0.2_)_3_, achieved a steady-state photocurrent of 5.13 × 10^−5^ A—3.5 times higher than that of the reference device (SC-MAPbI_3_). Because the Pb-Br bond energy (~208 kJ/mol) exceeds that of Pb-I (~179 kJ/mol), the stability of the perovskite inorganic framework is enhanced by Br^−^ doping. Consequently, the formation energy of halide vacancies—which act as primary non-radiative recombination centers—is increased, thereby effectively suppressing the defect density. Furthermore, the valence band maximum (VBM) is shifted downward by the introduction of Br^−^ resulting in bandgap widening. This simultaneously adjusts the material’s spectral response range and, in certain cases, optimizes charge injection by improving energy level matching. Crucially, the intrinsic stability of the material is also enhanced. The average A-site radius in the mixed-cation system is effectively reduced by the introduction of a small amount of Cs^+^, thereby adjusting the tolerance factor t-value towards a more desirable range. This facilitates the release of lattice stress, fostering the formation of a cubic perovskite structure with reduced structural stress, enhanced symmetry, and a lower intrinsic defect density. Consistent with the TRPL results, non-radiative recombination is significantly suppressed through the stabilization of the lattice by the Cs^+^ introduction, which substantially prolongs the carrier lifetime and thereby ensures more efficient collection of photo-generated carriers. Crucially, as shown in Figure 6b, when devices are fabricated on flexible PEN substrates, their I-V characteristics are nearly identical to those of rigid devices. This demonstrates the exceptional substrate versatility of the process strategy developed in this work.

To further evaluate the dynamic response characteristics and operational stability of the detectors, transient current–time (I-t) tests were conducted under a periodically switched X-ray source. As shown in Figure 6b and Figure S1b, rapid and highly repeatable responses were observed by all devices across multiple switching cycles, demonstrating their excellent potential as detectors. The photocurrent intensity was fully consistent with the steady-state I-V results, with the Cs^+^/Br^−^ co-doped device achieving a maximum response current of approximately 5.5 × 10^−5^ A. Furthermore, undoped MAPbI_3_ devices exhibited slight current drift during illumination, which is attributed to ion migration under the electric field—a known bottleneck for perovskite device stability. In contrast, rectangular response profiles with negligible drift were displayed by the Br^−^ and Cs^+^ doped devices, demonstrating superior signal stability. This indicates that ion migration during operation is effectively suppressed by the Cs^+^/Br^−^ co-doping, which establishes a more robust lattice structure, thereby suppressing signal drift. Furthermore, when compared to the rigid device in Figure 6c, the flexible device exhibits highly consistent performance in photocurrent intensity, response speed, and signal stability. This retention of performance across rigid and flexible substrates demonstrates the excellent versatility of the process, thereby reaffirming the practical applicability of this strategy in the field of flexible electronics.

To evaluate the radiation hardness and long-term operational stability, the detectors were subjected to continuous X-ray irradiation for 60 s, with results presented in Figure 6c and Figure S1c. Under sustained irradiation, significant differences in the stability of films with different compositions were observed. As a control, the pure MAPbI_3_ device (black and red curves) exhibited a gradual increase in response current during the initial phase of irradiation before reaching a stable plateau. This initial current rise can be attributed to the combined effects of trap-state filling and electric-field-driven ion migration, which are phenomena characteristic of perovskite films containing a higher density of mobile defects. In contrast, highly stable response currents were maintained by the Cs^+^ and Cs^+^/Br^−^ co-doped devices throughout the entire 60-s test window, with no discernible decay observed. This demonstrates that the Cs^+^/Br^−^ co-doping strategy effectively enhances structural stability by reinforcing the lattice, thereby improving tolerance to ion migration and radiation damage. Furthermore, devices fabricated on flexible substrates exhibited stability patterns consistent with those on rigid substrates, demonstrating the versatility of this optimization strategy.

To quantitatively evaluate the enhancement of X-ray detection capability enabled by composition engineering, we systematically measured the photocurrent response of the devices under various dose rates and calculated their corresponding sensitivities. Figure 6d illustrates the current response curves of the rigid-substrate detectors across different dose rates. All devices exhibited excellent linear response characteristics (R_2_ > 0.99), indicating that the carrier generation and recombination processes within the material reached a dynamic equilibrium within the tested range. The sensitivity of the EV-MAPbI_3_ device was determined to be approximately 8375 μC⋅Gy_air_^−1^⋅cm^−2^. Upon the incorporation of Br^−^ ions, the sensitivity of MAPb(I_0.8_Br_0.2_)_3_ significantly increased to approximately 13,625 μC⋅Gy_air_^−1^⋅cm^−2^. Remarkably, the device optimized through synergistic Cs^+^/Br^−^ engineering, (Cs_0.05_MA_0.95_)Pb(I_0.8_Br_0.2_)_3_, achieved the highest sensitivity of 1.4 × 10^4^ μC⋅Gy_air_^−1^⋅cm^−2^. This represents a ~67% improvement compared to the pure tri-iodide composition, demonstrating the immense potential of the triple-cation/mixed-halide strategy in enhancing charge collection efficiency.

A similar performance enhancement trend was observed on the flexible PEN substrates (Figure S1d). The sensitivity of the flexible EV-MAPbI_3_ detector was 6530 μC⋅Gy_air_^−1^⋅cm^−2^, while the synergistically engineered flexible (Cs_0.05_MA_0.95_)Pb(IBr)_3_ device reached a peak sensitivity of 14,325 μC⋅Gy_air_^−1^⋅cm^−2^. Interestingly, the sensitivity of the flexible synergistic device was slightly higher than that of its rigid counterpart. This could be attributed to the SPVE process facilitating superior interfacial contact or generating fewer stress-induced defects on the flexible substrate. Such ultra-high sensitivity maintained in a flexible state renders these devices highly competitive for applications in wearable medical X-ray imaging and industrial non-destructive testing. This dramatic boost in sensitivity is highly consistent with the material characterization results: the ultra-long carrier lifetime (~3497.34 ns) confirmed by TRPL implies that photogenerated carriers possess longer diffusion lengths before recombination, thereby significantly enhancing the overall charge collection rate.

From the processing perspective, the SPVE method precisely modulates the diffusion and reaction kinetics of vacuum-evaporated organic components into the solution-processed inorganic precursor. This hybrid approach ensures full precursor conversion and high crystallinity at a low temperature (100 °C). This specific processing route yields a superior structure characterized by a highly dense, large-grained morphology (Figure 2) and optimized chemical states (Figure 5). As evidenced by the PL and TRPL results (Figure 4), this structure significantly reduces the density of charge-trapping sites and suppresses non-radiative recombination. Consequently, this leads to the observed high performance (Figure 6), including an exceptionally low dark current (5.2 nA cm^−2^) and a record-level sensitivity (1.43 × 10^4^ μC⋅Gy_air_^−1^⋅cm^−2^) at 0 V.

**Figure 7:**
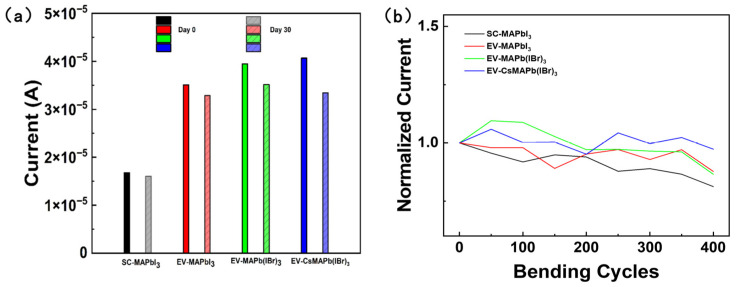
Environmental stability and mechanical flexibility testing of perovskite X-ray detectors with varying processes and compositions. (**a**) Comparison of X-ray response for devices with different compositions prepared via spin-coating (SC) and solution-processed vacuum evaporation (EV) before and after 30 days of storage in air. (**b**) Normalized photocurrent versus bending cycles (0–400 cycles) for different devices under a 6 mm bending radius.

To evaluate device reliability under practical operating conditions, the photoconductive response of perovskite detectors fabricated via different processes and compositions was compared before and after 30 days of air storage, as shown in Figure 7a. While slight performance degradation was observed across all devices, those fabricated via the EV process maintained robust response levels. Notably, the (Cs_0.05_MA_0.95_)Pb(I_0.8_Br_0.2_)_3_ device not only exhibited the highest initial current (4.1 × 10^−5^ A) but also retained a high current of 3.41 × 10^−5^ A after 30 days of storage. This indicates that the optimized devices maintain superior performance compared to the control samples, even after aging.

As flexible electronic devices, mechanical durability serves as a key metric for evaluating their performance. As illustrated in Figure 7b, the devices were subjected to 400 consecutive bending cycles at a 6 mm radius of curvature, recording the variation in normalized photocurrent. The reference sample SC-MAPbI_3_ exhibited the lowest mechanical stability, with its photocurrent retaining only 81.2% of the initial value after 400 bending cycles. This is attributed to the presence of numerous voids and grain boundaries in the spin-coated film, which act as stress concentration points and facilitate microcrack propagation during bending. EV-MAPbI_3_ and EV-MAPb(IBr)_3_ exhibited some resistance to bending, yet still suffered approximately 12–14% performance loss after 400 cycles (decreasing to 87.7% and 86.5%, respectively). In contrast, the optimized (Cs_0.05_MA_0.95_)Pb(I_0.8_Br_0.2_)_3_ device demonstrated exceptional mechanical robustness, retaining 97.3% of its initial photocurrent after 400 bending cycles with minimal fluctuation. This exceptional flexibility stems from the high density and reduced grain boundaries of the optimized film, which effectively dissipate mechanical stress and suppress crack formation during bending.

## 3. Experimental Details

Flexible PEN/ITO conductive substrates were purchased from Oike Industry Co., Ltd. (Kyoto, Japan), exhibiting a sheet resistance of 15 Ω/sq. PEDOT:PSS aqueous solution (1.3–1.7 wt% in H_2_O) was procured from Xi’an Yuri Solar Co., Ltd. (Xi’an, China). Lead iodide (PbI_2_, 99.9%) was obtained from TCI Shanghai Chemical Co., Ltd. (Shanghai, China). Methylammonium hydroiodide (MAI, >99.99%) was purchased from Greatcell Solar Materials (Queanbeyan, Australia). Lead bromide (PbBr_2_, 99.999%) was purchased from Aladdin (Riverside, CA, USA), and cesium iodide (CsI, 99.9%) was obtained from Alfa Aesar (Ward Hill, MA, USA). 2,2′,2″-(1,3,5-benzenetriyl)tri(1-phenyl-1H-benzimidazole) (TPBi, >99%) was purchased from Macklin (Shanghai, China). Dimethylformamide (DMF, 99.8%) and dimethyl sulfoxide (DMSO, 99.8%) were both purchased from Aladdin. Isopropyl alcohol (IPA, >99%) was purchased from Alfa Aesar. Concentrated hydrochloric acid (HCl, 36–38%) for ITO etching and other analytical-grade solvents (e.g., ethanol, acetone) were obtained from Sinopharm Chemical Reagent Co., Ltd. (Shanghai, China). Lithium fluoride (LiF) was purchased from RHAWN (Philadelphia, PA, USA). All chemicals were used without further purification.

First, the PEN/ITO substrates were chemically etched with hydrochloric acid to define the desired electrode pattern. Subsequently, the etched substrates were sequentially ultrasonicated for 30 min each in a dilute detergent solution, a ternary solvent mixture (ethanol, isopropanol, and acetone; 1:1:1, *v*/*v*/*v*), and absolute ethanol, with air-drying between each step (Shanghai Kedao, Shanghai, China, SK3300H). The cleaned substrates were immediately subjected to 30 min of surface treatment within an ozone-UV treatment unit (Peking University, 3.2 KW). Within a nitrogen-atmosphere glove box (Shanghai Micaelona, Shanghai, China), PEDOT:PSS solution was filtered through a 0.22 μm aqueous filter (Bikeman Biotechnology Co., Ltd., Changde, China) and spin-coated onto the ITO surface at 4000 rpm for 30 s (Kunshan Lidian, Kunshan, China, KW-4A). Subsequently, the samples were annealed for 20 min at 100 °C in a constant-temperature drying cabinet (Gaoxiang Industrial Equipment, Shengzhou, China, B15.U-157) to form the HTL layer. PbI_2_ (0.691 g) was dissolved in a mixed solvent comprising 900 μL DMF and 100 μL DMSO, magnetically stirred for 8 h. For the halogen-doped and cation-engineered films, PbBr_2_ and CsI were used as additional precursors. For MAPb(IBr)_3_, the precursor solution was prepared by mixing PbI_2_ and PbBr_2_ in a molar ratio of 7:3. For CsMAPb(IBr)_3_, 5% molar ratio of CsI (relative to the total Pb content) was further added to the above solution. Notably, for these doped solutions, the solvent mixture of DMF and DMSO was adjusted to a volume ratio of 8:2 to ensure complete dissolution and optimal film morphology. All other fabrication steps, including MAI evaporation and annealing, remained identical to those used for the pure MAPbI_3_ films. The precursor solution was spin-coated onto the HTL four times at 4000 rpm, followed by annealing on a 70 °C hot plate for 10 min. Subsequently, the samples were transferred to a high-vacuum thermal evaporation system (Suzhou Fangsheng Optoelectronics, Suzhou, China, FS450-S12). Upon achieving a chamber vacuum below 5 × 10^−4^ Pa, MAI was thermally evaporated at a rate of 0.3 Å/s to form a 580-nm-thick layer. Finally, the samples were annealed for 30 min at 100 °C in nitrogen atmosphere to complete the perovskite layer preparation. The resulting perovskite films were re-loaded into the vacuum thermal evaporation system. TPBi was sequentially evaporated at 0.2 Å/s to a thickness of 80 nm, followed by 1 nm of LiF and 150 nm of Al to form the top electrode.

The surface and cross-sectional morphologies were examined using scanning electron microscopy (SEM, ZEISS Sigma 360, ZEISS, Oberkochen, Germany). The crystal structure was analyzed via X-ray diffraction (XRD, SmartLab, Seoul, Republic of Korea). Photoluminescence (PL) spectra and time-resolved PL decay curves were measured using an FLS1000 spectrophotometer (Edinburgh Instruments, Livingston, UK) with a 475 nm excitation wavelength. Curve fitting was subsequently performed using a biexponential function to determine the fluorescence lifetime values. X-ray photoelectron spectroscopy (XPS) spectra of the perovskite films were characterized using a Thermo Scientific K-Alpha instrument (Thermo Fisher Scientific, Waltham, MA, USA). The current–voltage (I-V) characteristics of the devices were measured using a Keithley 2400 digital source meter (Tektronix, Beaverton, OR, USA). X-ray detection performance was tested using an X-ray generator (NDT160 K500 W05 MR X-ray source, Shenzhen Xinjiechu Mechanical Technology Co., Ltd., Shenzhen, China), with the response signal recorded by the aforementioned digital source meter. For the 30-day stability test, the encapsulated devices were stored in darkness at room temperature, 25 °C, with an ambient relative humidity of 45% ± 5%.

For material characterizations including SEM, XRD, XPS and optical spectroscopy (PL/TRPL), perovskite films with a thickness of ~300 nm (prepared via a single-cycle PbI_2_ deposition) were used to ensure clear surface/interface analysis. For the final X-ray detector fabrication, the thickness was scaled up to ~1 μm via a 4-cycle deposition process to optimize X-ray attenuation, while maintaining the same evaporation and annealing parameters to ensure consistent film quality.

## 4. Conclusions

In this work, a low-temperature solution-processed vacuum evaporation method was developed and optimized to fabricate high-quality perovskite films on flexible substrates, overcoming the inherent limitations of poor crystallinity and high defect density common in conventional solution methods. Systematic compositional engineering revealed that while bromine (Br^−^) incorporation enhances device sensitivity, synergistic co-doping with cesium (Cs^+^) yields the optimal overall performance. The optimal flexible detector, based on (Cs_0.05_MA_0.95_)Pb(I_0.8_Br_0.2_)_3_, demonstrated a remarkably low dark current density of 5.2 nA cm^−2^ and a high X-ray sensitivity of 1.43 × 10^4^ μC·Gy_air_^−1^·cm^−2^. Furthermore, the device maintained excellent mechanical stability with over 95% of its initial performance after 400 bending cycles (6 mm radius). This synergy between the optimized deposition process and compositional engineering provides a feasible technical solution for the development of low-cost, high-performance flexible X-ray detectors for portable medical and industrial imaging.

## Figures and Tables

**Table 1:** Comparison of performance metrics between our Solution-processed vacuum evaporation-processed flexible X-ray detector and other reported perovskite-based detectors.

Material System	Substrate	Method	Thickness (μm)	Sensitivity (μC·Gy_air_^−1^·cm^−2^)	Ref.
MAPbI_3_	Rigid	Blade-coating	830	1.1 × 10^4^ (240 V mm^−1^)	[41]
CsPbBr_3_	Rigid	Screen-printing	>300	1.6 × 10^4^ (80 V mm^−1^)	[42]
BA_2_MA_2_Pb_3_I_10_	Rigid	Spin-coating	10	214 (500 V mm^−1^)	[43]
CsPbI_2_Br	Rigid	Spray-coating	40	1.48 × 10^5^ (125 V mm^−1^)	[44]
CsPbI_2_Br/CsPbIBr_2_	Rigid	Vapor deposition	30	2.6 × 10^4^ (50 V)	[45]
(BZA)_2_CsPb_2_Br_7_	Flexible	Mechanical exfoliation	12	693 (0 V)	[46]
NH_3_(CH_2_)_4_NH_3_BiI_5_	Flexible	Membrane casting	~100	7872 (50 V)	[47]
(MDABCOBr)NH_4_(BF4)_3_	Flexible	Single crystal	-	2377 (200 V mm^−1^)	[48]
CsMAPb(IBr)_3_	Flexible	Solution-processed vacuum evaporation	1	1.43 × 10^4^ (0 V)	This work

**Table 2:** Fluorescence decay lifetime parameters of PVK films prepared by different processes and compositions.

Sample	τ_1_ (ns)	A_1_ (%)	τ_2_ (ns)	A_2_ (%)	τ_ave_ (ns)
SC-MAPbI_3_	610.69	66.38	5969.94	33.62	874.63
EV-MAPbI_3_	609.79	73.77	5580.46	26.23	795.71
EV-MAPb(IBr)_3_	709.22	77.03	6234.97	22.97	890.46
EV-CsMAPb(IBr)_3_	1649.28	38.78	12,050.87	61.22	3497.34

## Data Availability

The data that support the findings of this study are available from the corresponding author upon reasonable request.

## Supplementary Materials

The following supporting information can be downloaded at: https://www.mdpi.com/article/10.3390/molecules31071123/s1. Figure S1. (a) I-V characteristics, (b) dynamic response to X-ray irradiation, (c) continuous irradiation for 60 seconds, and (d) linear behaviour of photocurrent as a function of incident dose rate for perovskite devices on PEN substrates. All devices were encapsulated with PDMS to isolate moisture and oxygen; Figure S2. Statistical distribution of (a) dark current density and (b) X-ray sensitivity for 10 independent rigid (black) and 10 independent flexible (red) detectors; Figure S3. Optical characterization of the perovskite thin films. (a) UV-Vis absorption spectra of MAPbI_3_, MAPb(IBr)_3_, and CsMAPb(IBr)_3_ films. (b) Corresponding Tauc plots derived from the absorption data; Figure S4. Surface elemental analysis of the pristine MAPb(IBr)_3_ perovskite film. (a–c) EDS mapping images showing the spatial distribution of (a) Pb, (b) I, and (c) Br elements; Figure S5. Surface elemental analysis of the CsMAPb(IBr)_3_ perovskite film. (a–d) EDS mapping images showing the spatial distribution of (a) Pb, (b) I, (c) Br, and (d) Cs elements; Figure S6. Original TRPL decay curves and bi-exponential fitting reports for (a) SC-MAPbI_3_, (b) EV-MAPbI_3_, (c) EV-MAPb(IBr)_3_, and (d) EV-CsMAPb(IBr)_3_. Each panel includes the raw data points (yellow), the fitting curve (cyan), and the corresponding residuals plot (magenta) at the bottom; Figure S7. SCLC curves of the devices(ITO/PEDOT:PSS/Perovskite/Au). The trap-filled limit voltage (VTFL) values are 2.32 V (black), 0.81 V (red), and 0.26 V (green), respectively; Table S1. EDS quantitative analysis of the MAPb(IBr)_3_ reference film; Table S2. EDS quantitative analysis of the CsMAPb(IBr)_3_ reference film.
