# Supplementary material for: High-Quality Perovskite Films Enabled by Solution-Processed Vacuum Evaporation for Flexible PIN-Type X-Ray Detectors
Source: Molecules. 2026 Mar 29;31(7):1123. doi: 10.3390/molecules31071123 (PMC13074683; doi:10.3390/molecules31071123)
Supplement: Supplementary file 1 [file molecules-31-01123-s001.zip › molecules-4166907-supplementary.pdf]

High-Quality Perovskite Films Enabled by Solution-Assisted Vacuum Evaporation for Flexible PIN-Type X-Ray Detectors

Yali Wang[1], Hongjun Mo[1], Sai Huang[1], Haonan Li[1], Xinyang Huang[1], Weiguang Yang[*,1,2,3]

1. School of Materials Science and Engineering, Shanghai University, Shanghai 200444, China
2. Zhejiang Institute of Advanced Materials, SHU, Jiashan 314113, China
3. State Key Laboratory of Advanced Special Steels, Shanghai 200444, China

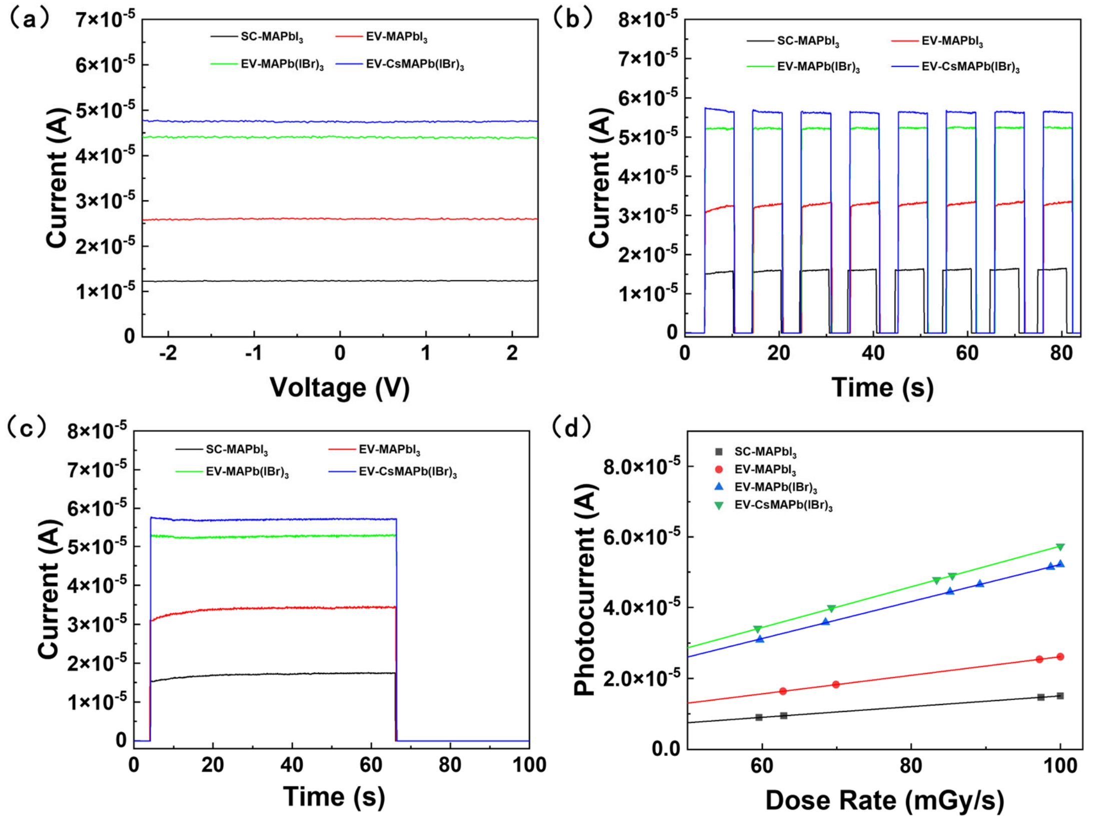

Figure S1 (a) I-V characteristics, (b) dynamic response to X-ray irradiation, (c) continuous irradiation for 60 seconds, and (d) linear behaviour of photocurrent as a function of incident dose rate for perovskite devices on PEN substrates. All devices were encapsulated with PDMS to isolate moisture and oxygen.

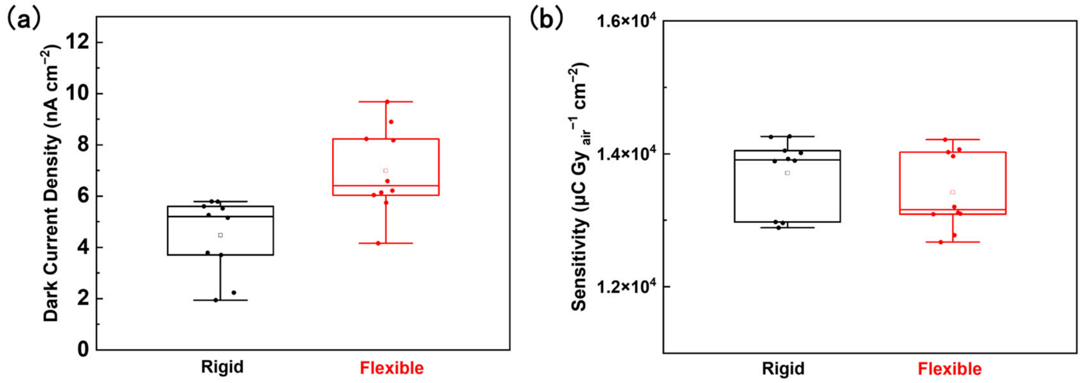

Figure S2. Statistical distribution of (a) dark current density and (b) X-ray sensitivity for 10 independent rigid (black) and 10 independent flexible (red) detectors.

As shown in Figure S2(a), the dark current densities for both rigid and flexible devices remained consistently low (approximately 5–10 nA cm^{-2}), with no significant deviation between the two groups. This indicates that the SPVE process facilitates the formation of high-quality film with low defect density regardless of the substrate type.

Furthermore, the X-ray sensitivity distribution is presented in Figure S2(b). Both rigid and

flexible detectors exhibited high sensitivities, with average values of approximately 1.36×10^4 μC Gy_{air}^{-1} cm^{-2}. The narrow distribution (coefficient of variation < 5%) and the high consistency between rigid and flexible samples further underscore the robustness of our fabrication strategy. These results confirm that the reported high performance is highly reproducible and that the flexible devices do not suffer from performance degradation compared to their rigid counterparts.

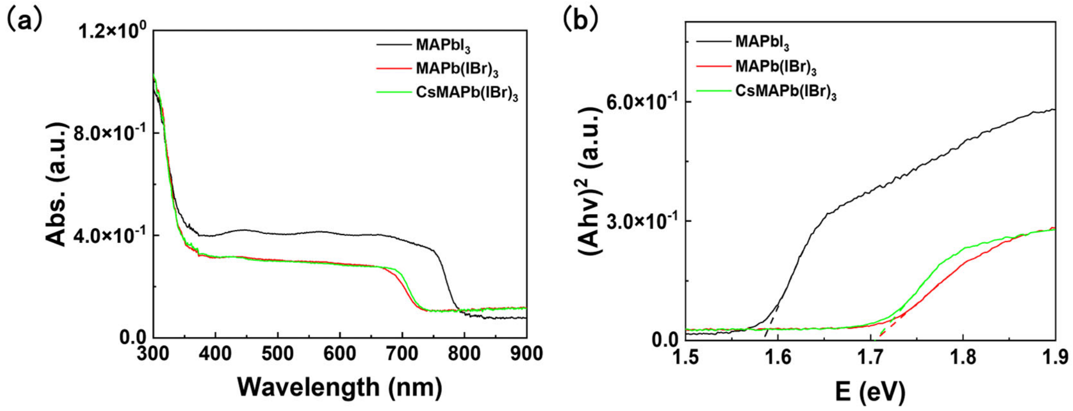

Figure S3. Optical characterization of the perovskite thin films. (a) UV-Vis absorption spectra of $MAPbI_3$, $MAPb(IBr)_3$, and $CsMAPb(IBr)_3$ films. (b) Corresponding Tauc plots derived from the absorption data

As shown in Figure S3(a), a significant blue-shift of the absorption edge from ~780 nm ($MAPbI_3$) to ~730 nm ($MAPb(IBr)_3$ and $CsMAPb(IBr)_3$) is observed. This shift directly corresponds to the widening of the optical bandgap (Eg) from 1.58 eV to 1.70/1.71 eV, as calculated from the Tauc plots in Figure S3(b). These results provide conclusive evidence that the incorporation of Br and Cs induces a genuine modification of the perovskite band structure, rather than solely improving film morphology.

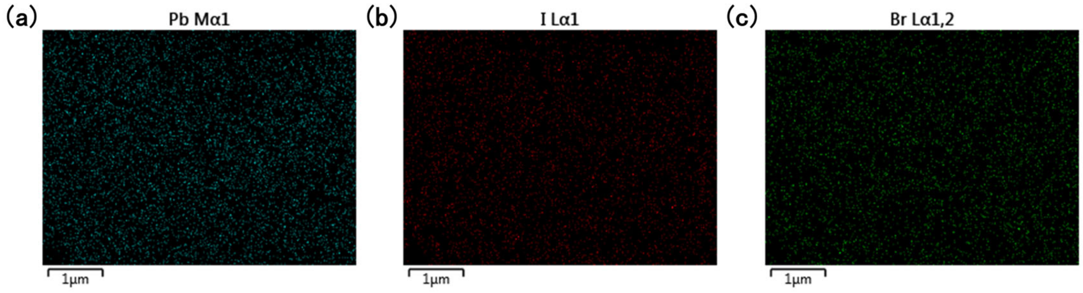

Figure S4 Surface elemental analysis of the pristine $MAPb(IBr)_3$ perovskite film. (a–c) EDS mapping images showing the spatial distribution of (a) Pb, (b) I, and (c) Br elements.

Table S1 EDS quantitative analysis of the $MAPb(IBr)_3$ reference film

Element	Line Type	Weight %	Wt % Sigma	Atomic %
I	L series	41.76	0.78	23.05
Cs	L series	0.00	1.01	0.00
Br	L series	12.25	0.38	10.74
C	K series	9.22	0.36	53.78

Pb	M series	36.77	0.81	12.43
Total		100.00		100.00

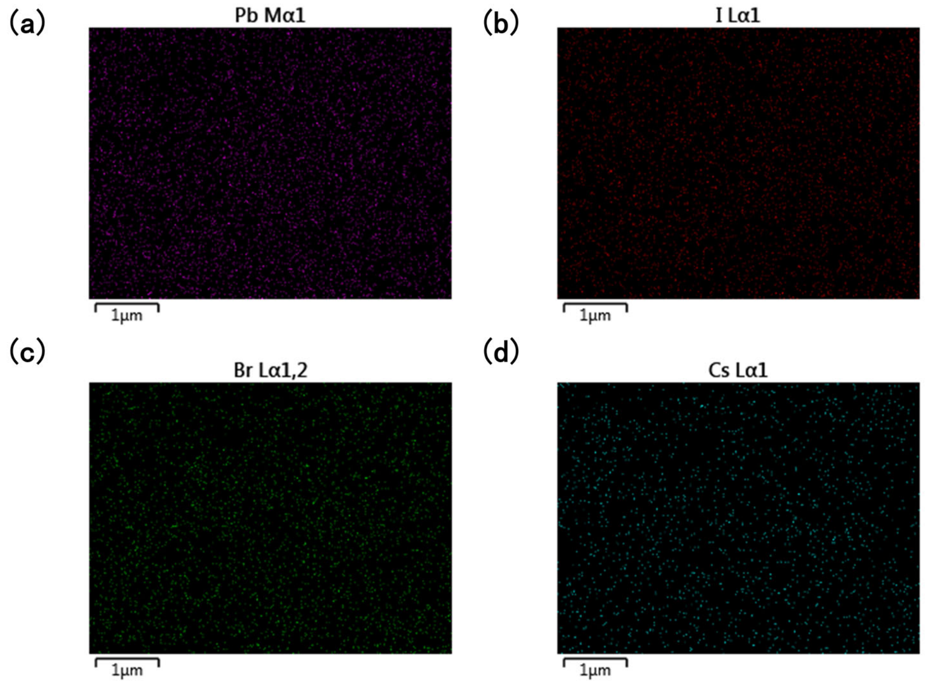

Figure S5 Surface elemental analysis of the $CsMAPb(IBr)_3$ perovskite film. (a–d) EDS mapping images showing the spatial distribution of (a) Pb, (b) I, (c) Br, and (d) Cs elements.

Table S2 EDS quantitative analysis of the $CsMAPb(IBr)_3$ reference film

Element	Line Type	Weight %	Wt % Sigma	Atomic %
I	L series	43.74	0.72	26.14
Pb	M series	38.44	0.72	14.07
C	K series	8.04	0.29	50.79
Br	L series	9.01	0.28	8.55
Cs	L series	0.76	0.81	0.43
Total		100.00		100.00

The synergistic integration of cation and anion engineering in the perovskite films was systematically verified through EDS mapping and quantitative analysis. As illustrated in Figures S4 and S5, both the $MAPb(IBr)_3$ and the $CsMAPb(IBr)_3$ films exhibit exceptional elemental homogeneity across the entire scanned micro-scale regions. No evidence of elemental segregation or secondary phase formation (such as CsI or $PbBr_2$ clusters) was observed, indicating that the dopants are uniformly incorporated into the perovskite lattice.

Quantitative results summarized in Tables S1 and S2 further confirm the successful modulation of the chemical composition. The pristine film (Table S1) shows a baseline Br content of 10.74 at% with zero Cs presence. In contrast, the experimental film (Table S2) reveals the successful incorporation of Cs (0.43 at%) alongside Br (8.55 at%).

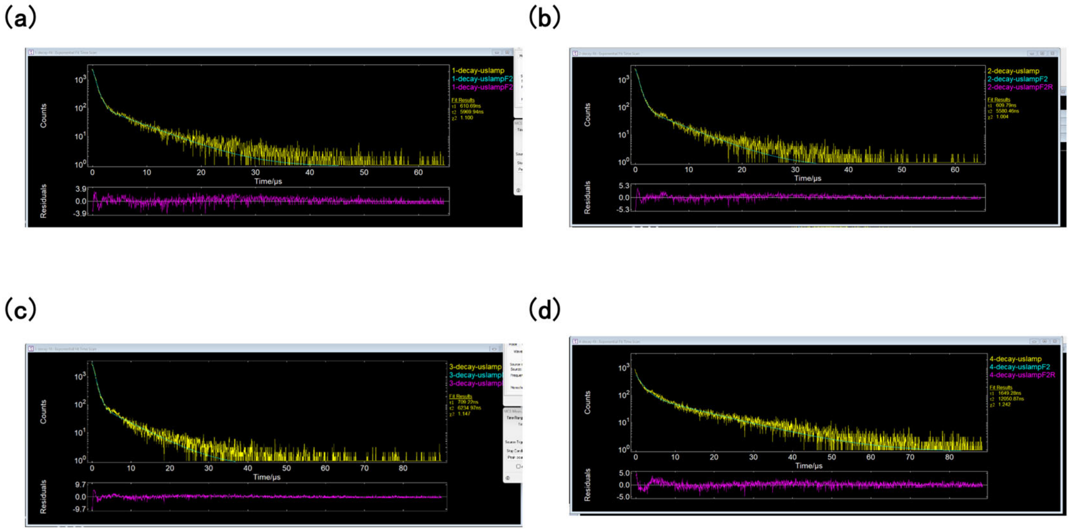

Figure S6 Original TRPL decay curves and bi-exponential fitting reports for (a) SC-$MAPbI_3$, (b) EV-$MAPbI_3$, (c) EV-$MAPb(IBr)_3$, and (d) EV-$CsMAPb(IBr)_3$. Each panel includes the raw data points (yellow), the fitting curve (cyan), and the corresponding residuals plot (magenta) at the bottom.

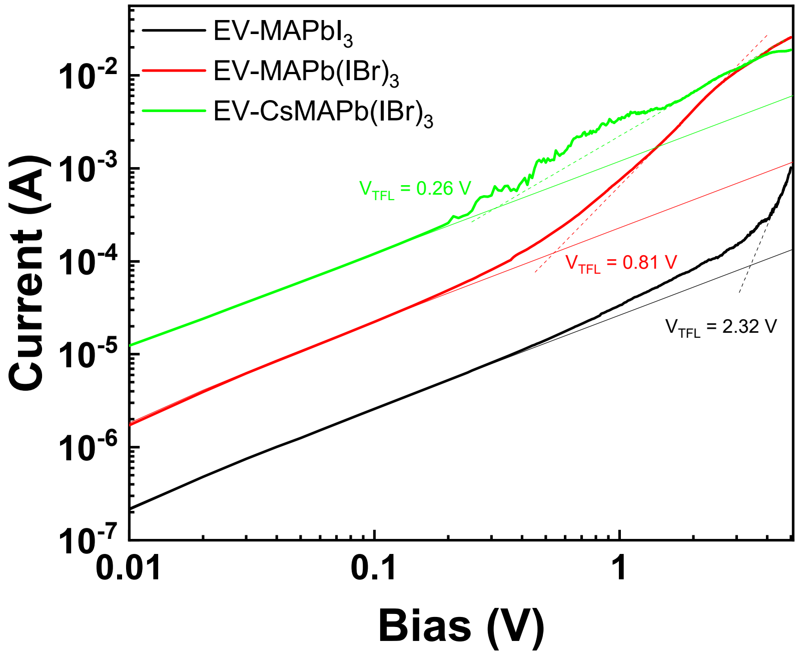

Figure S7 SCLC curves of the devices(ITO / PEDOT:PSS / Perovskite / Au). The trap-filled limit voltage (VTFL) values are 2.32 V (black), 0.81 V (red), and 0.26 V (green), respectively.

The extracted V_{TFL} decreased from 2.32 V (pure) to 0.26 V (Cs/Br doped), confirming a nearly ten-fold reduction in trap-state density, which aligns perfectly with the extended TRPL lifetimes.
